# Sustained delivery of osteogenic growth peptide through injectable photoinitiated composite hydrogel for osteogenesis

**DOI:** 10.3389/fbioe.2023.1228250

**Published:** 2023-08-08

**Authors:** Beibei Liu, Jiannan Wu, Xiaodi Sun, Qingxun Meng, Jian Zhang

**Affiliations:** ^1^ Department of Oral Implantology, Tianjin Stomatological Hospital, School of Medicine, Nankai University, Tianjin, China; ^2^ Key Laboratory of Oral and Maxillofacial Function Reconstruction, Tianjin, China

**Keywords:** OGP, GelMA, HAMA, bone tissue engineering, photo-crosslinking, sustained release

## Abstract

One of the most challenging clinical issues continues to be the effective bone regeneration and rebuilding following bone abnormalities. Although osteogenic growth peptide (OGP) has been proven to be effective in promoting osteoblast activity, its clinical application is constrained by abrupt release and easily degradation. We developed a GelMA/HAMA dual network hydrogel loaded with OGP based on a combination of physical chain entanglement and chemical cross-linking effects to produce an efficient long-term sustained release of OGP. The hydrogel polymers were quickly molded under ultraviolet (UV) light and had the suitable physical characteristics, porosity structure and biocompatibility. Significantly, the GelMA/HAMA-OGP hydrogel could promote cell proliferation, adhesion, increase osteogenic-related gene and protein expression *in vitro*. In conclusion, the OGP sustained-release system based on GelMA/HAMA dual network hydrogel offers a fresh perspective on bone regeneration therapy.

## 1 Introduction

Many individuals experience structural integrity loss of bone tissue out of trauma or illness each year (Zhang et al., 2019). Autologous bone grafting and allogeneic bone grafting are common traditional methods for the treatment of bone defects (Qing et al., 2020). However, their practical applicability is frequently constrained by the scarcity of autologous bone and immunological rejection of allogeneic bone (Safari et al., 2021). Metals, bioceramics, and polymers have all been researched as potential alternatives to bone grafts substitutes in order to overcome these problems (Bose et al., 2012). These scaffolds still have a number of drawbacks, though, including a lack of bioactivity, difficulty in cell attachment or incongruity between the rate of degradation and osteogenesis (Haugen et al., 2019). To address these issues, tissue engineering offers the prospect of more possibilities for bone tissue regeneration and repair (Rezwan et al., 2006). Simply put, to achieve efficient bone tissue repair, we can perform a three-dimensional scaffold with active growth factors and cells. This scaffold is able to release active components, mimicking the structure and repair process of the tissue, thus helping the bone tissue to recover quickly (Bhattacharjee et al., 2017; Wubneh et al., 2018; Wei et al., 2020).

As one of the important scaffold materials, hydrogels are three-dimensional reticular cross-linked scaffolds that can highly mimic extracellular matrix and promote cell viability, adhesion, and proliferation (Li et al., 2022; Xue et al., 2022). Among them, gelatin, as a hydrolysis product of natural collagen, contains many active functional groups such as amino and carboxyl groups as well as hydroxyl groups, and has good biocompatibility and low antigenicity (Kurian et al., 2022). In particular, gelatin has many cell-specific binding sites, such as matrix metalloproteinase-sensitive degradation sites and Arg-Gly-Asp sequences, which can promote cell adhesion, migration and differentiation (Zhang Y. et al., 2022). However, gelatin has a rapid degradation rate and poor mechanical properties. Therefore, gelatin was chemically modified with methacrylic acid to form methacrylated hydrogels (GelMA) (Yue et al., 2015). However, GelMA can not be used for an extended period of time because it is still a somewhat unstable substance that breaks down quickly in the absence of cells. Therefore, we added hyaluronic methacrylate (HAMA) to form a double network (DN) hydrogel to improve viscosity and stability. Hyaluronic acid (HA) is a prevalent component of the extracellular matrix that interacts with many cell surface receptors, such as cell adhesion molecule and intracellular adhesion molecule-1 (Zhai et al., 2020). In addition, the carboxyl and hydroxyl groups of hyaluronic acid can be chemically modified by methacrylates to form HAMA to further improve their chemical and mechanical properties, providing high levels of rigidity and rather elastic properties (Schuurmans et al., 2021). However, HA-based hydrogels are not conducive to cell adhesion, which hinders cell proliferation (Graça et al., 2020). GelMA is an excellent match for this deficit. Therefore, the development of natural composite hydrogels is an effective way to satisfy both cellular activity and mechanical properties.

However, only biological scaffolds do not have satisfactory osteogenic induction. One of the focuses of tissue engineering efforts is to investigate how growth factors can be associated with biological scaffolds to enhance their osteoconductivity and osteoinductivity. OGP is a natural molecule containing a highly conserved 14 amino acid motif. Studies have demonstrated that combining OGP with a biological scaffold enhances its role as an osteogenic inducer. Osteoblast differentiation, proliferation, and alkaline phosphatase activity are all successfully induced by OGP. In the meantime, OGP controls the transforming growth factor (TGF), insulin-like growth factor (IGF), and basic fibroblast growth factor (BFGF) to stimulate bone production and improve trabecular bone density *in vivo* (Pigossi et al., 2016; Policastro and Becker, 2016). Moreover, the amino acid sequence of OGP extracted from human serum is identical to that of OGP from rats and mice and is homologous to most animals, showing that the structure of OGP is evolutionarily conserved (Policastro and Becker, 2016). Therefore, we considered incorporating OGP into hydrogel scaffolds to increase its osteogenic activity. Currently, most studies have focused on delivery by physical adsorption or self-assembly. However, OGP is very sensitive to the surrounding environment and the non-covalent linkage is easily degraded on the scaffold surface (Mendes et al., 2013). Therefore, we attempted to graft and copolymerize OGP in GelMA/HAMA dual network hydrogels by photo-crosslinking to achieve effective long-term release of OGP.

In this study, an injectable photocrosslinkable dual-network composite hydrogel encapsulating OGP was developed to achieve *in situ* bone regeneration. The hydrogel introduces photocrosslinkable OGP that can click react with the methacrylic acid groups of GelMA and HAMA under UV exposure resulting in graft copolymerization. The combination of physical chain entanglement and chemical linkage effects of the dual network hydrogel ensures the long-term sustained release of OGP, creating a growth factor-rich microenvironment for bone repair and bone regeneration. We characterized the plasticity, injectability, pore-like morphology, swellability, degradability, rheological properties, and release rate of the biomaterial. The bioactivity was observed using CCK-8 and Calcein/PI staining and cell adhesion was examined using FITC-Phalloidin staining. Their osteogenic bioactivity was observed by alkaline phosphatase activity, alizarin red staining, RT-PCR and immunofluorescence staining. We concluded that GelMA/HAMA-OGP hydrogel could be a promising biomaterial for bone tissue regeneration.

## 2 Materials and methods

### 2.1 Preparation of GelMA/HAMA-OGP hydrogel

The freeze-dried prepolymers of GelMA and HAMA (Mw = 12–14 kDa, StemEasy, China) were fully dissolved in PBS (pH = 7.4) containing NHS and EDC under magnetic stirring at 50°C with the final concentrations of the polymers being 10% and 2% (w/v), respectively, to create the GelMA/HAMA double network hydrogels (O’Connell et al., 2016; Fan et al., 2020). Added photoinitiator 2-hydroxy-4’-(2-hydroxyethoxy)- 2-methylpropiophenone (1% w/v), in an addition reaction with photocrosslinkable OGP (MA-YGFGG, Qiangyao Company, China), GelMA/HAMA-OGP were photocrosslinked to create stable co-crosslinked hydrogels, with a final concentration of 10^–13^ M. Finally, the solution is cured for 30 s to construct hybrid crosslinking network hydrogel by being exposed to UV light.

### 2.2 Characterization of GelMA/HAMA and GelMA/HAMA-OGP hydrogel

Gelation was shown using a tabletop technique. Simply put, the physical state of a hydrogel sample in a vial is tested by turning the vial, replicating the effect of injecting the hydrogel into the body *in situ* at physiological temperatures.

In order to evaluate the shape plasticity of the hydrogel, we observed whether the hydrogel could be solidified into the desired shape by using different shapes of molds.

To evaluate the injectability of the hydrogel, the double network hydrogel was added to a syringe and extruded through an 18-gauge needle (*φ* ≈ 1.20 mm) to observe the state of extrusion.

And, the gelation time was determined by the inversion method. The initial time point when the hydrogel was flowless in the inverted bottle at room temperature was recorded in triplicate.

The porosity of the hydrogels was determined by the ethanol displacement method. The initial weight W_0_ and initial volume V_0_ of the samples were recorded. The samples were soaked in anhydrous ethanol for 5 min. After removal, the residual ethanol was wiped from the sample surface and the weight W_1_ was recorded. The density of ethanol was ρ. The porosity was calculated as: $porosity\;\left(\%\right)=\left(W_{1}-W_{0}\right)\;/\;\rho V_{0}\times 100\%\;\left(n=3\right)$ .

The scanning electron microscopy (SEM, Hitachi TM4000Plus, Japan) was used to examine the morphology of the GelMA/HAMA and GelMA/HAMA-OGP hydrogel scaffolds with a 15 kV acceleration voltage. The samples were lyophilized for SEM after being dehydrated in gradient ethanol and treated with 2.5% glutaraldehyde. Before SEM examination, platinum was sputter-coated onto the scaffold’s surface. There were three samples in each group. Five images were chosen from each sample at random.

Electrospray ionization mass spectrometry (ESI-MS) and high-performance liquid chromatography (HPLC) were used to analyze the OGP-MA.

GelMA, GelMA-OGP, HAMA, HAMA-OGP were characterized by Fourier transform infrared spectroscopy (FTIR, Nicolet iS10, United States). The hydrogels were first frozen at −80°C overnight, lyophilized, crushed and mixed with potassium bromide. The samples were then made into discs using a tablet press. Crushed samples ranging from 400 to 4,000 cm^−1^ were used for analysis with a resolution of 4 cm^−1^. 32 scans of the sample were performed for each FTIR measurement and the spectra obtained were the average of all scans.

A rheometer (MCR92, Anton Par) was used to investigate the hydrogels’ rheological characteristics to observe the change in viscosity with temperature, shear force and frequency respectively. Shear rates spanning 0.1–100 s^−1^ were used to examine the viscosity properties. The temperature sweep test measured viscosity at temperatures ranging from 10 to 60°C. The fluctuation of storage modulus (G′) and loss modulus (G″) with frequency (ω; rad/s) allowed for the observation of frequency sweep.

To study the swelling rate of GelMA/HAMA and GelMA/HAMA-OGP hydrogels, they were weighed after freeze-drying and marked with an initial weight of W_0_. The dried hydrogel samples were then immersed in PBS at room temperature for 0.5, 1, 2, 3, 4, 5, 6, 7 and 8 h. The mass of the wet hydrogel was recorded at each time point as W_t_. Swelling Ratio (SR) was calculated as: *SR=*(*W*_*t*_ *-W*_*0*_)*/W*_*0*_ *× 100%*.

To evaluate the degradation rate of GelMA/HAMA and GelMA/HAMA-OGP hydrogels, they were weighed after freeze-drying and labeled with an initial weight of W_0_. The freeze-dried samples were immersed in PBS containing 2.6 U/ml collagenases at 37°C in a humidified 5% CO_2_ atmosphere for 1, 2, 3, 5 and 7 days, then freeze-dried, weighed, and labeled with a weight of W_t_. Degradation Ratio (DR) was calculated as: *DR=*(*W*_*0*_ *-W*_*t*_)*/W*_*0*_ *× 100%*.

To plot the release profile of OGP in the hydrogel materials, we collected soaked supernatants of GelMA/HAMA-OGP hydrogels on 1, 3, 5, 7, and 14 days. The BCA protein concentration assay kit (Solarbio, China) was used to determine total protein concentrations.

### 2.3 Biocompatibility assays

The commercially available pre-osteoblast cell line MC3T3-E1 (National Infrastructure of Cell-line Resource, China) was cultured in MEM Alpha basic medium (α-MEM, Gibco, United States) containing 10% FBS (Gibco, United States) and 1% penicillin and streptomycin (Gibco, United States) at 37°C and 5% CO_2_.

The Cell Counting Kit-8 (CCK-8, Solarbio, China) assays was applied for cell viability. The hydrogels were cured in 48-well plates, sterilized with 75% ethanol for 30 min, then UV irradiated for 1 h, and then washed with PBS for 5 min with three changes of PBS. Then the MC3T3-E1s cells were seeded onto hydrogel surface with a density of 2×10^3^ cells/200 μL per well. After culture for 1, 3, 5, and 7 days, a 200 μL media containing 20 μL of the CCK-8 staining solution was added to each well. The plates were incubated for 2 hours at 37°C in an incubator with 5% CO_2_ humidity (Thermo Fisher Scientific, United States). Then, a microplate spectrometer (Tecan, Switzerland) was used to detect the absorbance intensity at 450 nm.

Live/Dead staining was carried out using Live/Dead Cell test kit (Beyotime, China). Firstly, MC3T3-E1s suspension with a density of 1×10^3^ was inoculated into 24-well tissue culture plates and incubated for 24 h. Cells were put to each well 72 h and stained with caledrin AM/PI. To distinguish between living and dead cells, add 250 μL of the cell detection reagent working solution to each well. 30 min incubation at 37°C protected from light was performed. Images were then taken with an inverted fluorescence microscope (Nikon, Japan).

In addition, cellular adhesion to the hydrogel composites was also evaluated. 1 × 10^4^ cells per well were inoculated on GelMA/HAMA and GelMA/HAMA-OGP hydrogels in 24-well plates. The actin protein was stained with FITC-Phalloidin (Beyotime, China) for 20 min and the nuclei were re-stained with 4′,6-diamidino-2-phenylindole (DAPI, Beyotime, China) for 3 min. The morphology and adhesion of attached cells were examined under fluorescence microscopy.

### 2.4 *In vitro* osteogenic differentiation and gene expression

The MC3T3-E1s cells were seeded into 6-well plates with a density of 1×10^5^ cells per well. When cell confluence reached 70%–80%, the complete medium was replaced with osteogenic differentiation medium. Osteogenic induction medium contained α-MEM, 10% FBS, 1% penicillin/streptomycin, 10 nM dexamethasone (Sigma-Aldrich, United States), 10 mM β-glycerophosphate (Sigma-Aldrich, United States), and 0.2 mM ascorbic acid (Sigma-Aldrich, United States). The GelMA/HAMA and GelMA/HAMA-OGP hydrogel scaffolds were soaked in osteogenic medium for 48 h to obtain extracts, which was then used to further incubate the cells for an additional 48 h.

For the ALP activity assay, cells were washed with PBS after 7 days of osteogenic induction, followed by lysis of cells and collection of cell lysates. The micro-BCA protein assay kit (Beyotime, China) was used to measure the total intracellular protein content before adjusting for ALP activity. The next step was to determine the ALP activity using an alkaline phosphatase test kit (Beyotime, China) in accordance with the manufacturer’s instructions. The absorbance readings were calculated using a microplate spectrometer and measured at 405 nm.

BCIP/NBT alkaline phosphatase staining kit (Beyotime, China) was used in accordance with the manufacturer’s instructions to carry out the ALP staining. Briefly, 6-well plates of MC3T3-E1 induced for 7 days were fixed with 95% ethanol, flushed with PBS buffer, and treated with 500 μL dye. The cells were then washed with PBS and imaged using an Olympus microscope and an EPSON scanner.

For Alizarin Red S (ARS) staining, cells were preserved with 95% ethanol for 15 min after 14 days of osteogenic induction. Cells were then gently rinsed three times with PBS buffer and stained for 5 min with 1% Alizarin Red S (pH = 4.2, Solarbio, China). Finally, the unbound dye was gently washed off the cell surface with PBS buffer. Under an inverted microscope, the cells were examined for orange-red mineralized nodules and documented. Subsequently, 10% cetylpyridinium chloride (CPC, Solarbio, China), which dissolves alizarin red in the presence of calcium deposition, was added to the alizarin red-stained culture plates. The optical density was measured at 562 nm after the supernatant had been collected.

We employed RT-PCR to measure the expression of genes linked to osteogenesis, including RUNX family transcription factor 2 (*RUNX2*), alkaline phosphatase (*ALP*), osteoblast-specific genes (*Osterix*), as well as osteocalcin (*OCN*) and osteopontin (*OPN*). Total RNA from the cells was extracted using the Mini BEST universal RNA Extraction Kit (TaKaRa, Japan) after 7 and 14 days of osteogenic induction, and concentrations were determined. The Prime Script RT kit (TaKaRa, Japan) was used for reverse transcription. By using TB Green Premix Ex Taq IIRT-PCR (TaKaRa, Japan), the cDNA template was amplified. As an internal reference control, GAPDH was utilized. Table 1 displays the primer sequences utilized for PCR amplification. A Light Cycler/Light Cycler 480 system from Roche (Switzerland) was used to perform RT-PCR.

**TABLE 1:** Primer sequences used for RT-PCR.

Gene	Forward primer (5′→3′)	Reverse primer (3′→5′)
RUNX2	CCG​AAA​TGC​CTC​CGC​TGT​TAT​G	GGA​TTT​GTG​AAG​ACT​GTT​ATG​GT
ALP	TAT​GTC​TGG​AAC​CGC​ACT​GAA​C	CAC​TAG​CAA​GAA​GAA​GCC​TTT​GG
OCN	GGT​GCA​GAC​CTA​GCA​GAC​ACC​A	AGG​TAG​CGC​CGG​AGT​CTA​TTC​A
OPN	AGC​AAG​AAA​CTC​TTC​CAA​GCA​A	GTG​AGA​TTC​GTC​AGA​TTC​ATC​CG
Osterix	CGT​CCT​CTC​TGC​TTG​AGG​AA	TTT​CCC​AGG​GCT​GTT​GAG​TC
GAPDH	CAGCAACTCCCACTCTTC	TGT​AGC​CGT​ATT​CAT​TGT​C

To evaluate the expression of osteogenic proteins, RUNX2 antibodies (Proteintech, China), Osterix (Abcam, United States) and OPN antibodies (Proteintech, China) were used to label target proteins. During the inoculation process, 500 cells per well were inoculated into 6-well plates for 7 days. The cells were immobilized with 4% paraformaldehyde for 15 min, and then penetrated with 0.5% Triton X-100 solution for 10 min. After blocking non-specific antigens incubated at 4°C overnight with primary staining buffer (Beyotime, China), the cells were then immersed in secondary staining buffer at room temperature for 45 min. The nucleus is labeled with DAPI to locate the cell. Fluorescent images are captured under an inverted fluorescence microscope in a dark environment.

### 2.5 Statistical analysis

Statistical analysis was conducted by using GraphPad Prism 5.0 software (San Diego, United States). The mean and standard error of the mean were used to represent all data. To ascertain the statistical difference between the two groups, a two-tailed Student’s *t*-test was applied. Data from more than two groups were compared using the one-way ANOVA method. Statistics were deemed significant at *p* < 0.05.

## 3 Results

### 3.1 Preparation and characterization

**FIGURE 1:**
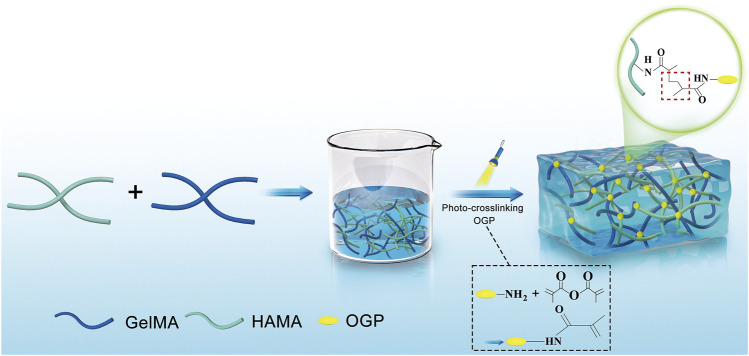
Schematic illustration of the fabrication procedures of the GelMA/HAMA-OGP hydrogels.

**FIGURE 2:**
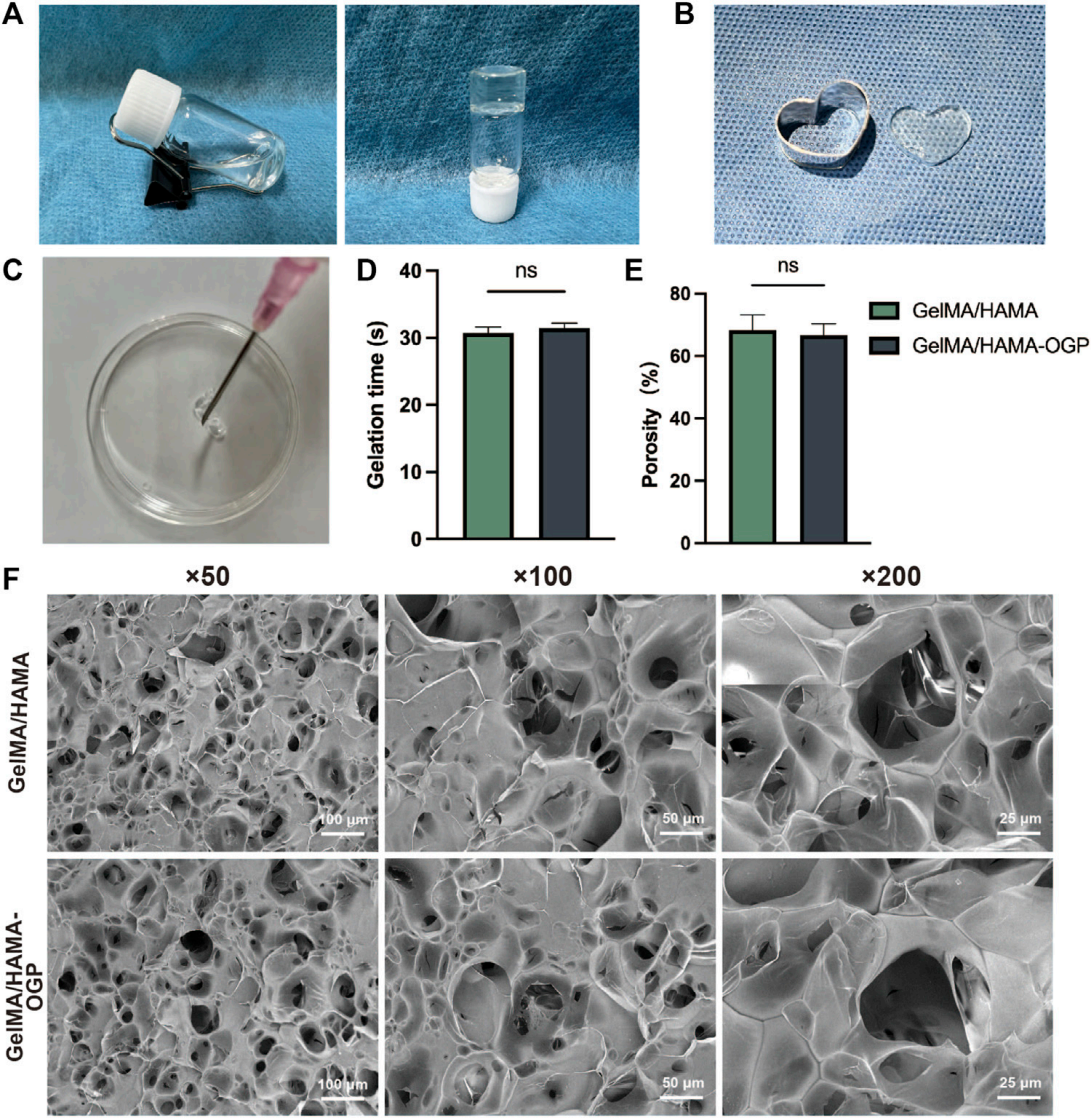
**(A)** Transformation of sol to gel after UV exposure. **(B)** The hydrogel can be made into different shapes of hydrogel materials in different modulus. **(C)** The GelMA/HAMA-OGP hydrogel injected via an 18-gauge needle (*φ* ≈ 1.20 mm). **(D)** Gelation time of GelMA/HAMA and GelMA/HAMA-OGP hydrogels. **(E)** Porosity of GelMA/HAMA with or without OGP loading. **(F)** SEM images of the porous structure of GelMA/HAMA and GelMA/HAMA-OGP. (ns, no significant difference).

Figure 1 depicts the steps in the production of GelMA/HAMA-OGP hydrogels. After exposure to UV light, the liquid GelMA/HAMA-OGP could transform into a gel-like solid at fast rate (Figure 2A). The hydrogel can be made into different shapes of hydrogel materials by using different shapes of molds (Figure 2B). The shear thinning behavior of the hydrogel demonstrated its excellent injectability and its ability to pass through the 18-gauge needle smoothly, facilitating the clinical implementation of the injection procedure (Figure 2C). Gelation time was not significantly different between GelMA/HAMA and GelMA/HAMA-OGP hydrogels, both of which could be rapidly light-cured and molded in a short time, demonstrating that the cross-linking of OGP did not affect the curing of the hydrogels (Figure 2D). The porosities of GelMA/HAMA and GelMA/HAMA-OGP hydrogels were 68.2% and 66.7%, respectively. The two groups did not significantly differ from one another (Figure 2E). A suitable porosity facilitates material exchange and is a necessary property for an ideal biomaterial (Loh and Choong, 2013; N’Diaye et al., 2013). The morphology of the hydrogels was observed by SEM technique (Figure 2F), and it is clear that there are no appreciable differences in the internal structures of GelMA/HAMA and GelMA/HAMA-OGP. This indicates that our encapsulation of OGP proteins in GelMA/HAMA dual network hydrogels did not change their structures. These hydrogel materials have appropriate pore size and provide favorable physical conditions for three-dimensional cell growth and material exchange (Lee and Shin, 2007). In contrast to conventional single-component hydrogels (Gu et al., 2018), small and large pores of dual-network hydrogels exist simultaneously and are dotted and connected to each other. This structure is crucial for biological behavior, as previous studies have demonstrated that smaller pores facilitate cell anchoring and adhesion, and larger pore sizes facilitate cell proliferation and spreading (Tang et al., 2016). Thus, it appears that GelMA/HAMA-OGP hydrogels allow for cell entry, internal and external circulation of nutrients, and metabolic waste efflux.

**FIGURE 3:**
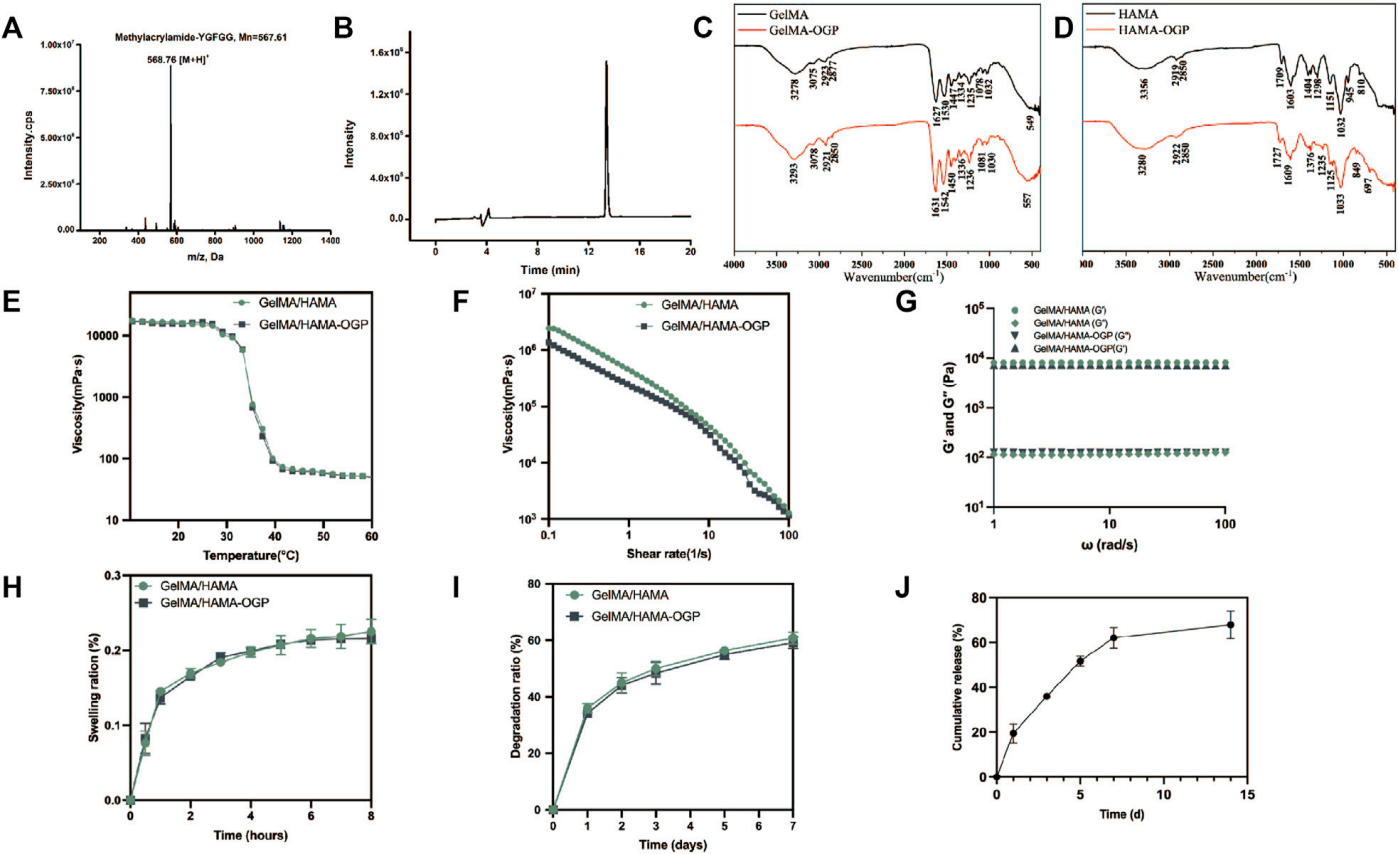
**(A)** The ESI-MS spectrum of OGP. **(B)** HPLC profiles of OGP with high purity (>99%). **(C)** FTIR spectra of GelMA and GelMA-OGP. **(D)** FTIR spectra of HAMA and HAMA-OGP. **(E)** Temperature-dependent viscosity ranging from 10°C to 60°C for different hydrogel. **(F)** Shear-rate-dependent viscosity ranging from 0.1 to 100 s^−1^ for different hydrogel composites at 37°C. **(G)** The fluctuation of storage modulus (G′) and loss modulus (G″) with frequency (ω; rad/s) allowed for the frequency sweep. **(H)** Swelling, **(I)** Degradation profiles of GelMA/HAMA, with or without OGP loading. **(J)** Cumulative release of OGP from the GelMA/HAMA-OGP hydrogel.

The peptide was characterized by ESI-MS and its molecular structure was determined (Figure 3A). The [M + H]^+^ of OGP was found at 568.76 Da, consistent with its theoretical molecular weight at 567.61 Da. HPLC analysis showed a high purity of 99.23% for the peptide (Figure 3B). These results demonstrate the successful synthesis of OGP-MA. In the FTIR spectra of GelMA-OGP, compared to GelMA, they all produced absorption peaks at the same position with only minor absorption peak migration, indicating that the peptides have the same groups after modification. However, compared to GelMA spectra, its absorption peaks at 1,631, 1,542 and 1,236 cm^−1^ were enhanced, where the absorption peaks were mainly caused by C=O, C-N and N-H groups. These indicate that the peptide OGP grafted to GelMA hydrogel caused the enhancement of absorption peaks at the same position, so the peptide successfully modified GelMA material (Figure 3C). In the FTIR spectrogram of HAMA-OGP, its absorption peak at 1,298 cm^−1^ becomes faint compared to HAMA, while a new absorption peak appears at 1,235 cm^−1^, where the absorption peak is related to the peptide N-H bending vibration. Meanwhile, the absorption peaks of C=O stretching vibration near 1,750∼1,400 cm^−1^ were all shifted to some extent, which were related to the peptide C=O structure. All these indicate that OGP interacts with HAMA correlatively, generating covalent bonding and grafting on HAMA successfully (Figure 3D). The rheological performance of hydrogel materials was characterized for the effects of shear, temperature and frequency on the viscosity. The viscosity profile of this hydrogel varied linearly at room temperature, independent of temperature, with more stable production conditions. By increasing the temperature, the fluidity of the hydrogel increases significantly, facilitating the injection operation (Figure 3E). These hydrogel solutions exhibit a similar diminishing trend with increasing shear rate and are viscous in the low shear rate range. When the shear rate approached 10 s^−1^ or greater, the viscosity substantially falled, indicating that they are injectable and malleable, which facilitates bone repair operations during treatment (Figure 3F). The storage modulus (G′) and loss modulus (G″) of GelMA/HAMA and GelMA/HAMA-OGP hydrogel tended to be stable in the scan range, and over the frequency range, the energy storage modulus was above the loss modulus, indicating that the mesh structure of hydrogel maintains a stable gel state throughout the test range (Figure 3G). The dilatability of hydrogels is strongly related to its network structure, mechanical strength, and capacity to govern the transport of cellular nutrients and metabolites, in addition to its capacity to absorb bodily fluids. The swelling curves showed that GelMA/HAMA and GelMA/HAMA-OGP hydrogels almost reached the equilibrium swelling state after 5 h of immersion in PBS, with the swelling ratios of 22.5% ± 1.9%, 21.6% ± 0.5%, respectively (Figure 3H). The rate at which the osteogenic scaffold material degrades is another crucial characteristic since it should coincide with the rate at which new bone tissue is forming (Koons et al., 2020; Wei et al., 2020). We employed collagenases to examine the biodegradation behavior of this hydrogel because they are abundant in the physiological milieu of humans and are crucial for biomaterials to *in vivo* breakdown (Wu et al., 2021). The degradation experiments showed that the degradation rates of the hydrogels were 60.8% ± 0.7%, 59.2% ± 1.6% after 7 days of incubation in PBS containing collagenases, with very similar degradation trends (Figure 3I). In addition, we also examined the *in vitro* release of OGP loaded in GelMA/HAMA-OGP hydrogels. As shown in Figure 3J, the cumulative release profile showed that the initial burst of OGP release occurred within 24 h, accounting for approximately 18% of the overall amount. The initial abrupt release was able to effectively stimulate cell proliferation and differentiation in the bone defect area (Fanaee et al., 2021). OGP protein release was observed for up to 14 days, which is due to the synergistic effect of physical encapsulation and chemical interlocking of the dual network hydrogel to ensure sustained drug release. The release rate of OGP gradually slowed down with time, and the cumulative release of OGP at 14 days was about 70%, which indicates that GelMA/HAMA-OGP hydrogel has sustained release properties and provides a stable drug delivery system. The slow release of OGP is beneficial to maintain the effective concentration of extracellular drugs in the scaffold to promote bone tissue regeneration and repair sufficiently and durably (Jayaraman et al., 2015).

### 3.2 Biocompatibility assays

**FIGURE 4:**
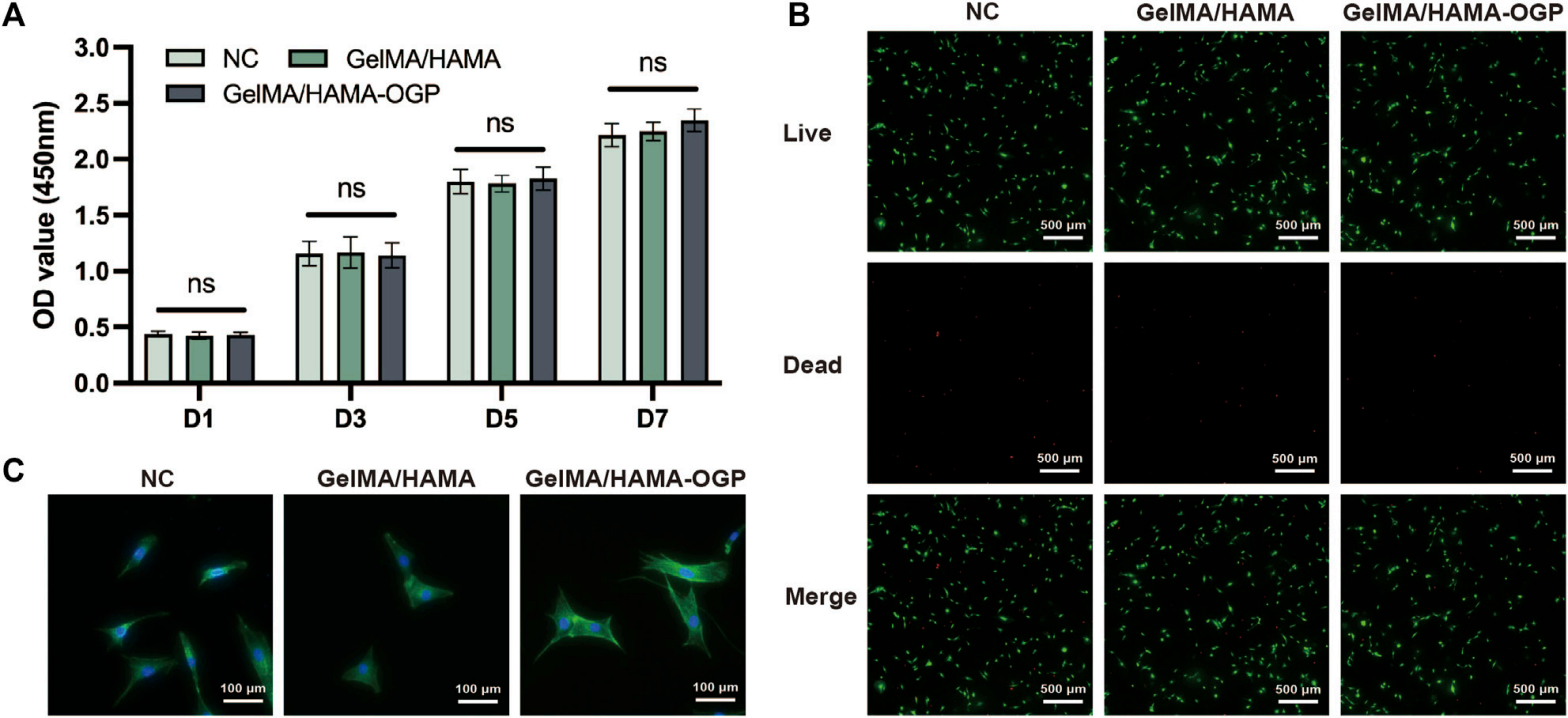
**(A)** Cell viability of MC3T3-E1 after cultivated on three groups for 1,3, 5 and 7 days evaluated by CCK-8 assay. (ns, no significant difference). **(B)** Calcein AM/PI staining: Green (Calcein AM) shows live cells, Red (EthD-1) shows dead cells. Scale bar = 500 μm. **(C)** MC3T3-E1 attachment and spreading behavior on different hydrogel samples was assessed by phalloidin and DAPI staining.

The cytocompatibility of the hydrogels was assessed by cell viability and cell adhesion. We firstly demonstrated the biosafety of GelMA/HAMA-OGP hydrogels by CCK-8 and live-dead staining assays. The three groups did not significantly differ from one another, and all three groups of cells increased with time (Figure 4A). The experimental results showed that GelMA/HAMA-OGP composite hydrogel has great biocompatibility and biosafety. Calcein AM/PI staining was used to distinguish live and dead cells. Most of the stained cells were alive and green in color, and only a few red fluorescent spots appeared (Figure 4B). In addition, most of the cells showed polyhedral morphology, which indicated that MC3T3-E1 could survive, spread, and adhere well on the gel surface, and the hydrogel composite provided a suitable biological microenvironment.

Early cell adhesion is critical for subsequent cell differentiation and directly affects subsequent biological behavior (Shuai et al., 2018). To investigate cell expansion and morphology, the cytoskeleton was stained and photographed after MC3T3-E1 was cultured for 24 h on various hydrogels. MC3T3-E1 adhered to the hydrogel surface in a polygonal structure, exhibiting a well-stretched morphology and extending more contact pseudopods (Figure 4C). These findings imply that the hydrogel serves as a useful matrix for promoting cell adhesion and proliferation, and that integrin-mediated binding can improve the cell-matrix interaction between MC3T3-E1 and hydrogels by binding to the RGD motif in the gel matrix (Xiao et al., 2019). Moreover, the hydrogel’s cross-linked structure, appropriate viscoelasticity, and rough surface promote cell attachment (Li et al., 2018).

### 3.3 *In vitro* osteogenic differentiation and gene expression

**FIGURE 5:**
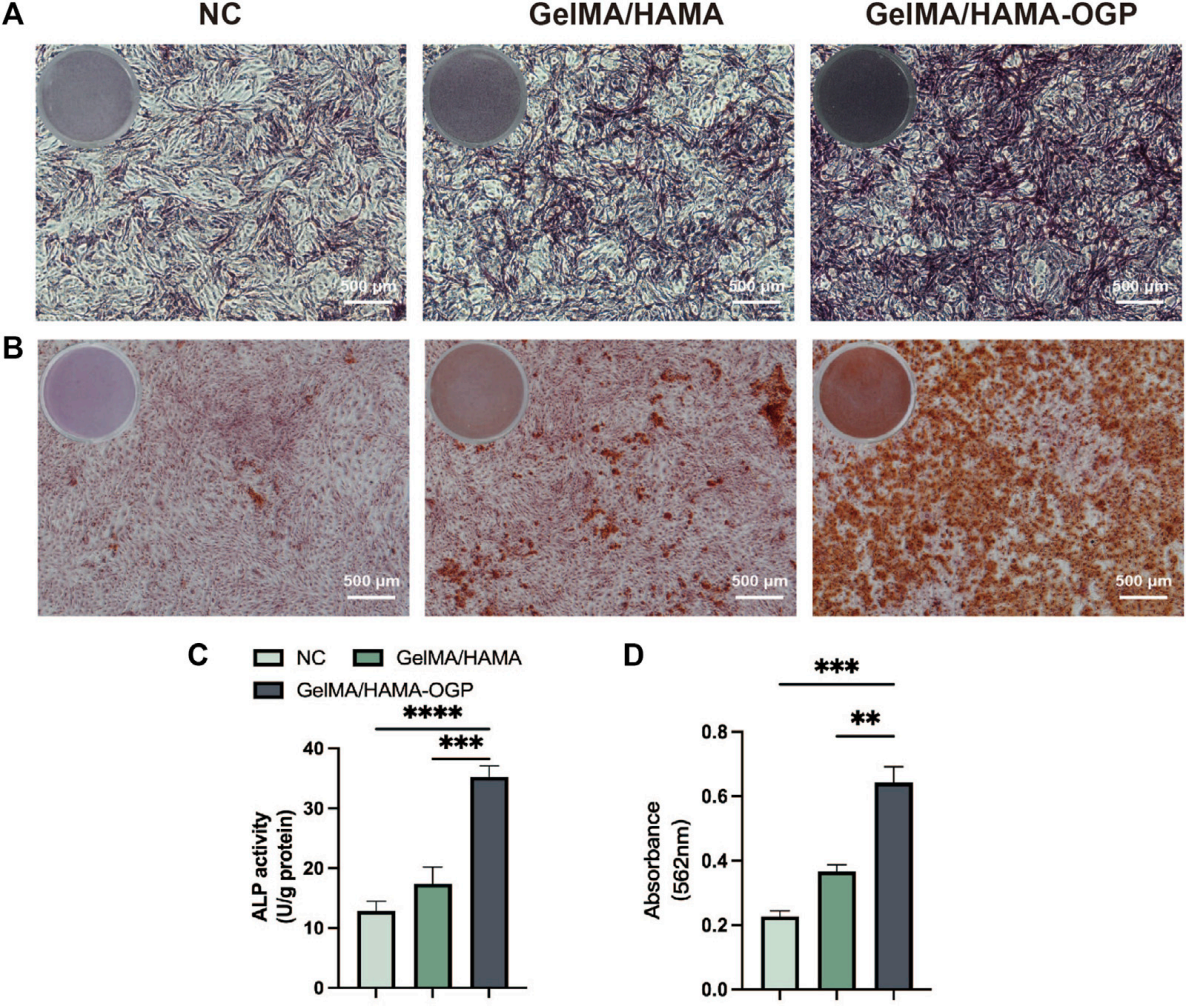
**(A)** Representative gross-micrographs of ALP staining on day 7. Scale bar = 500 μm. **(B)** Representative gross-micrographs of alizarin red staining on day 14. Scale bar = 500 μm. **(C)** Semi-quantitative analysis of ALP activity for 7 days. **(D)** Semi-quantitative analysis of alizarin red staining for 14 days. (**p < 0.01, ***p < 0.001, ****p < 0.0001)

Ideal osteogenic biomaterials should be effective at promoting cellular osteogenic differentiation together with strong biocompatibility. The osteogenic induction ability of GelMA/HAMA-OGP hydrogel scaffolds was evaluated by ALP staining, ARS staining, immunofluorescence staining and RT-PCR. ALP staining and ALP assay kits allow for the qualitative and quantitative analysis of ALP activity, which can be employed as an early marker of cellular osteogenic differentiation. Positive staining outcomes for the ALP assay were seen in all groups (Figure 5A). Notably, the GelMA/HAMA-OGP hydrogel group showed more positive staining results at day 7 compared to the control and GelMA/HAMA hydrogel groups. ALP activity was also measured further to assess the osteogenic development. The ALP activity survey showed that the ALP activity of both GelMA/HAMA-OGP group was significantly higher than that of the control group (****p < 0.0001) and GelMA/HAMA group (***p < 0.001) (Figure 5C). This result suggests that GelMA/HAMA hydrogel promotes MC3T3-E1 differentiation toward osteoblasts and the presence of OGP contributes to the upregulation of ALP. After 14 days of co-culture with hydrogel, MC3T3-E1 cells were stained with alizarin red to demonstrate calcium nodule development, a phenotypic hallmark of late osteogenic differentiation, which demonstrated the capacity for late osteogenic differentiation. The findings revealed that the blank group had tiny crimson nodules. The GelMA/HAMA group in the control group displayed an increase in red nodules, however the overall number was not significant. Numerous red mineralized nodules and robust positive red reaction were seen in the GelMA/HAMA-OGP hydrogel group (Figure 5B). Similar results were obtained when the OD values of mineralized nodules that were solubilized in CPC were quantified (Figure 5D). According to the aforementioned findings, MC3T3-E1 cells undergoing late osteogenic differentiation can considerably increase calcium deposition and mineralization in GelMA/HAMA-OGP hydrogel.

**FIGURE 6:**
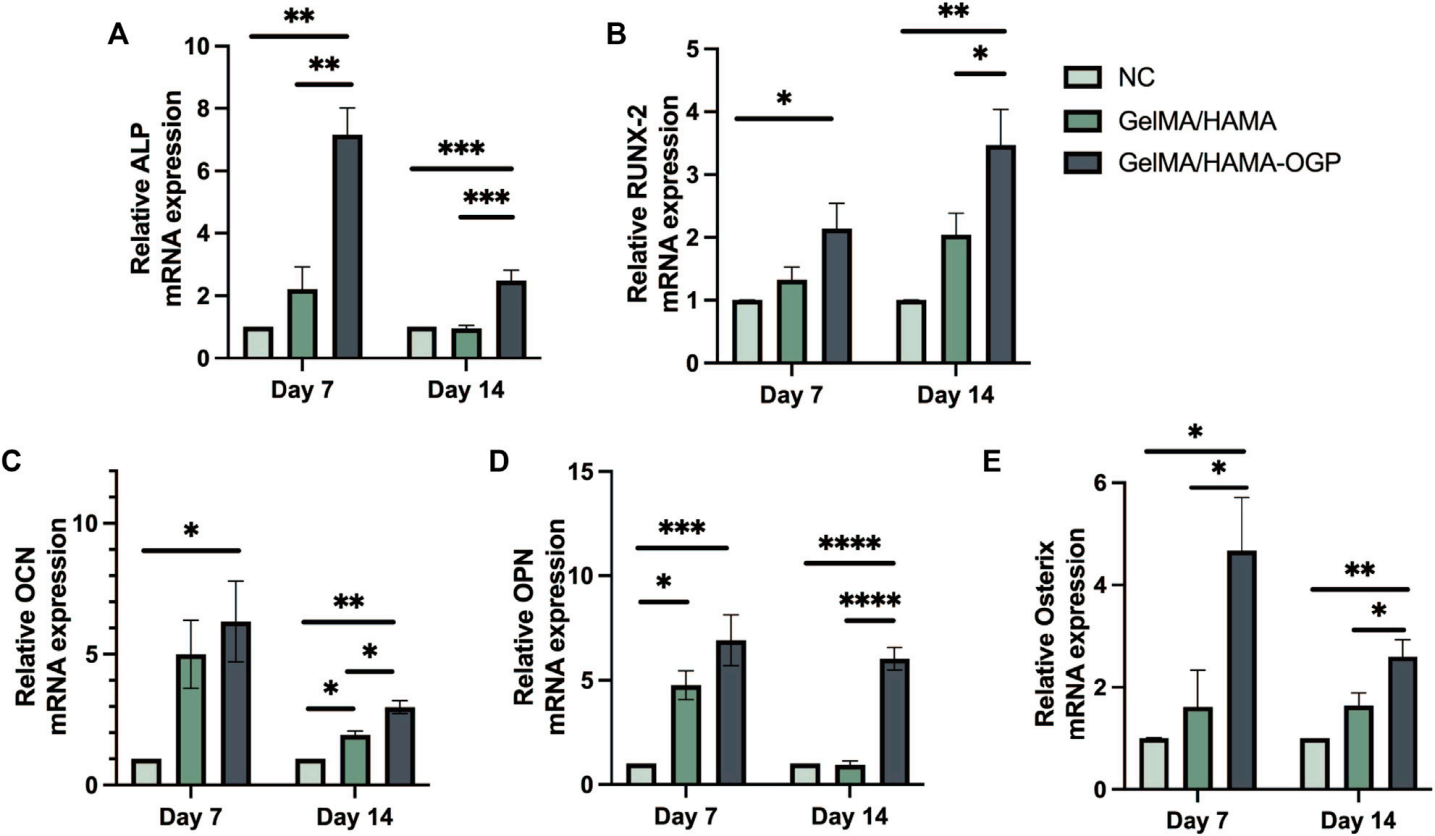
RT-PCR analysis of osteogenesis-related gene expressions including **(A)** *ALP*, **(B)** *RUNX2*, **(C)** *OCN*, **(D)** *OPN*, **(E)** *Osterix*. Statistically significant differences are indicated with *p < 0.05, **p < 0.01, ***p < 0.001, ****p < 0.0001.

To further explore the osteogenic differentiation of MC3T3-E1 cells on different samples, we use RT-PCR to detect the gene expression of osteogenic-related proteins after 7 and 14 days of induced osteogenesis, including *ALP* (early marker of osteogenic differentiation) (Manolagas, 2020; Vimalraj, 2020), *RUNX2* (central regulator of osteoblast differentiation (Wang et al., 2022)), *OCN* (regulates Ca^2+^ homeostasis and bone mineralization as a bone mineralization stage marker (Manolagas, 2020; Wei and Karsenty, 2015)), *OPN* (major bone non-collagen component (Rodan, 1995) and *Osterix* (transcriptional regulator involved in osteoblast development and bone regeneration (Sinha and Zhou, 2013)). As shown in Figures 6A–E, the expression of five genes associated with osteogenic differentiation was upregulated in the GelMA/HAMA-OGP group compared with other groups, confirming their osteogenic activity. In conclusion, the GelMA/HAMA-OGP group enhances the osteogenic ability of MC3T3-E1 cells *in vitro* and is a promising scaffold material to promote osteogenic differentiation.

**FIGURE 7:**
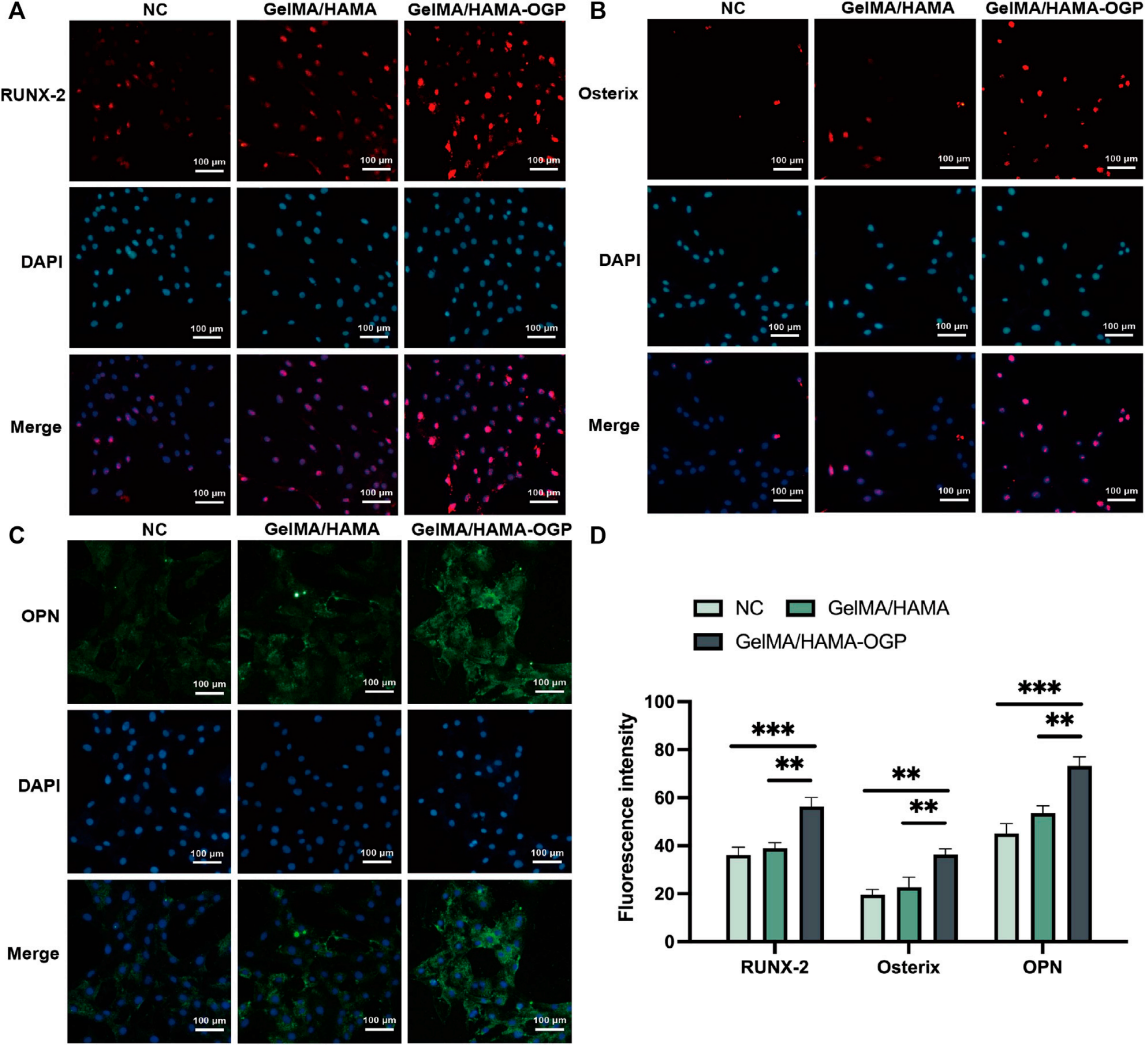
Representative immunocytochemistry images of osteogenesis-related protein including **(A)** RUNX-2, **(B)** Osterix and **(C)** OPN. **(D)** Semi-quantitative analysis of fluorescence intensity. Statistically significant differences are indicated with **p < 0.01, ***p < 0.001.

We used immunostaining assays to measure the synthesis of several osteogenic-related proteins after 7 days. Among the early indicators of osteoblast differentiation, RUNX2 regulates the transcription of genes encoding osteogenic-specific matrix proteins and modulates the expression of osteogenic-specific matrix protein genes in the MC3T3-E1 cell line (Yan et al., 2022). Osterix is a downstream gene of RUNX-2 and plays an important role in the induction of osteoblast differentiation and bone formation (Liu et al., 2020). As shown in Figures 7A, B, the expression of RUNX2 was evaluated by immunofluorescence staining on day 7. The levels of RUNX2 were higher in the GelMA/HAMA-OGP hydrogel group than in the control and GelMA/HAMA hydrogel groups, with an increased number of positive cells and enhanced immunofluorescence signal. In addition, OPN is a marker of middle to late osteogenic differentiationis and considered to be an important factor in bone remodeling (Depalle et al., 2021). Similarly, the GelMA/HAMA- OGP hydrogel scaffolds showed the greatest staining intensity and proportion of positive areas compared to the other two groups (Figure 7C). Among these three groups, the fluorescence intensity of various proteins (e.g., Runx2, Osterix and OPN) all in the GelMA/HAMA-OGP group showed the highest intensity (Figure 7D).

## 4 Discussion

In this study, we designed a hydrogel-based OGP slow-release system with photo-crosslinked injectable GelMA/HAMA dual networks. GelMA/HAMA-OGP hydrogel is capable of successfully promoting osteogenic differentiation and bone tissue regeneration. It also has good degradability, sustained release and biocompatibility properties, thus owns broad application prospects.

In recent years, researches on double network hydrogels that mimic natural tissues have flourished, overcoming the performance bottleneck of single network hydrogels. In contrast to single network hydrogels, DN hydrogels have an interpenetrating polymer network structure, which has a denser spatial network structure and improve mechanical characteristics, tunability, and workability (Vega et al., 2017). DN hydrogels typically consist of a hard and brittle first network and a flexible and ductile second network. The first network, which is brittle and unyielding, supports the entire system mechanically and has a high crosslink density. Because of its low crosslink density and “sacrificial bond” function, the second network’s flexibility and ductility enable the DN hydrogel to function as an energy dissipator while shielding the first network from harm during stressful situations (Nonoyama and Gong, 2021; Xu et al., 2021). Moreover, *in situ* gelation, which can quickly and easily fill irregular defects of any shape, is required for bone repair surgery (Bernhard and Tibbitt, 2021; Zhang et al., 2021). In this study, GelMA/HAMA-OGP was shown to have excellent flexibility, spatial and temporal control, and the ability to initiate gelation by one-step photo-crosslinking.

Biological factors are another key factor for successful osteogenesis, as the process of bone regeneration is intricate and involves numerous biological components. OGP, a significant growth factor connected to bone regeneration, has been thoroughly investigated and coupled with various biomaterials to support bone regeneration. OGP is initially extracted during the bone formation phase of bone marrow regeneration, a 14 amino acid peptide (NH2-ALKRQGRTLYGFGG-OH) (Bab, 1995). The complete sequence of OGP (10–14) is the smallest fragment that maintains full-length OGP activity, namely, Tyr10-Gly11-Phe12-Gly 13-Gly14 (Pigossi et al., 2016). OGP has a highly conserved amino acid sequence, which suggests that OGP in many mammals may have the same biological function. The amino acid sequence of OGP extracted from human blood is identical to that of rat and mouse OGP and is analogous to most animals (Kadam et al., 2007; Liu et al., 2019; Schmidt et al., 1997). OGP promotes proliferation, differentiation, ALP activity, and matrix mineralization, all of which are crucial for bone repair and regeneration. Additionally, OGP controls TGF, IGF, and BFGF expression, promoting *in vivo* bone growth and trabecular bone density. However, when physically loaded onto scaffolds, it is vulnerable to inactivation, burst release and uncontrolled diffusion, which can result in risks like cytotoxicity and high local drug concentrations. Hence, scientists have been trying to figure out how to release active OGP in a long-lasting manner. OGP was delivered via the drug carrier of DNA nano-framework nucleic acids thanks to Zhang T et al (T. Zhang et al., 2022). Liu Y et al. prepared a poly (l-lactic acid) nanofiber scaffold immobilized with OGP using a polydopamine (PDA)-encapsulated method, which can sustainably release OGP for improved osteogenesis (Liu et al., 2019). Gina M. et al. modified phenylalanine PEUs (poly(1-PHE-6)) by tethering OGP to tyrosine-based monomer subunits. By physical chain entanglement, our GelMA/HAMA-OGP dual-network hydrogel can encapsulate the drug and aid in gradual release (Policastro et al., 2015). Most crucially, the methacrylic acid groups of GelMA and HAMA create covalent connections with the photo-crosslinkable OGP, allowing for the gradual and continuous release of OGP to enhance osteogenesis.

While the hydrogel composite is a meaningful step towards promotion of bone regeneration, it also has several limitations. Firstly, in studying the physical properties of the hydrogel polymer, only the time point of its initial formation has been considered. However, it is unknown how cell growth and differentiation affect the hydrogel scaffold’s physical qualities. Secondly, the mechanical strength of the scaffold material is important for bone tissue regeneration in load-bearing areas or larger bone tissue defects. The present study failed to reveal the effect of mechanical properties of hydrogel scaffolds on bone tissue regeneration in detail. Future studies may explore this issue in more depth by preparing hydrogels with different mechanical strengths and repairing different types of bone defects.

## 5 Conclusion

In conclusion, we developed a photo-crosslinked injectable GelMA/HAMA dual network hydrogel-based OGP sustained-release system and showed that it had good biomechanical and biological activity to effectively induce osteogenesis. The hydrogel has an appropriate porosity structure, is capable of continuously releasing OGP for a predetermined amount of time, and possesses appropriate swelling, degradation, and rheological properties. Studies on cytotoxicity and cell proliferation revealed that MC3T3-E1 cells were not adversely affected by the hydrogel. The hydrogel demonstrated good biocompatibility and promoted cell attachment. Osteogenesis-related gene and protein were all upregulated *in vitro* by the GelMA/HAMA dual network hydrogel. The findings imply that GelMA/HAMA-OGP hydrogel has excellent application potential.

## Data Availability

The original contributions presented in the study are included in the article/supplementary material, further inquiries can be directed to the corresponding author.
